# Alternative treatment strategies to accelerate the elimination of onchocerciasis

**DOI:** 10.1093/inthealth/ihx054

**Published:** 2018-02-19

**Authors:** Michel Boussinesq, Grace Fobi, Annette C Kuesel

**Affiliations:** 1 IRD UMI 233-INSERM U1175-Montpellier University, 34394 Montpellier, France; 2 African Programme for Onchocerciasis Control, Ouagadougou, Burkina Faso; 3 UNICEF/UNDP/World Bank/WHO Special Programme for Research and Training in Tropical Diseases, Geneva, Switzerland

**Keywords:** Alternative treatment strategies, Elimination, Macrofilaricidal drugs, New treatments, Onchocerciasis, Test-and-Treat strategies

## Abstract

The use of alternative (or complementary) treatment strategies (ATSs) i.e. differing from annual community-directed treatment with ivermectin (CDTI) is required in some African foci to eliminate onchocerciasis by 2025. ATSs include vector control, biannual or pluriannual CDTI, better timing of CDTI, community-directed treatment with combinations of currently available anthelminthics or new drugs, and ‘test-and-treat’ (TNT) strategies requiring diagnosis of infection and/or contraindications to treatment for decisions on who to treat with what regimen. Two TNT strategies can be considered. Loa-first TNT, designed for loiasis-endemic areas and currently being evaluated using a rapid test (LoaScope), consists of identifying individuals with levels of *Loa* microfilaremia associated with a risk of post-ivermectin severe adverse events to exclude them from ivermectin treatment and in treating the rest (usually >97%) of the population safely. Oncho-first TNT consists of testing community members for onchocerciasis before giving treatment (currently ivermectin or doxycycline) to those who are infected. The choice of the ATS depends on the prevalences and intensities of infection with *Onchocerca volvulus* and *Loa loa* and on the relative cost-effectiveness of the strategies for the given epidemiological situation. Modelling can help select the optimal strategies, but field evaluations to determine the relative cost-effectiveness are urgently needed.

## Onchocerciasis control and elimination strategies to date

Three international programmes have supported countries to control onchocerciasis as a public health problem or to eliminate onchocerciasis: the Onchocerciasis Elimination Program for the Americas (OEPA), the Onchocerciasis Control Programme (OCP) in West Africa and the African Programme for Onchocerciasis Control (APOC). The OEPA was launched in 1993 to address onchocerciasis in 13 foci in Brazil, Colombia, Ecuador, Guatemala, Mexico and Venezuela (approximately 565 000 people in 2359 communities).^1,2^ Its strategy was health system–provided ivermectin mass drug administration (MDA). A variable number of annual treatments, followed by 17–25 biannual treatments and quarterly distributions (in approximately 460 communities), have resulted in confirmed or likely elimination in all but the large Amazonian focus spanning the Venezuela–Brazil border, with approximately 30 000 people at risk in 541 communities.^2–4^

In Africa, onchocerciasis elimination encounters significantly greater challenges: endemicity in 31 countries, a >100 times larger population at risk and conflicts in many countries. In West Africa, onchocerciasis control was initiated in 1974 through the OCP, which eventually covered 1.3 million km^2^ in 11 countries.^5^ The population at risk was 17.8 million, with 7.55 million infected.^6^ The primary strategy was vector control through weekly aerial larviciding of breeding sites along approximately 50 000 km of rivers.^7,8^ After MSD, also known as Merck & Co., Inc., Kenilworth, NJ, USA, decided in 1987 to donate ivermectin (Mectizan) for onchocerciasis control, ivermectin treatment complemented vector control in the Southern Extension and Western Extension and was the only control measure in some areas of the Western Extension.^7^ Ivermectin was distributed in collaboration with non-governmental development organizations (NGDOs)^9^ using mobile teams including local people.^5^ In 1996, a multicountry study showed that distribution systems designed by communities themselves (community-directed treatment with ivermectin [CDTI]) increase coverage.^10^

When OCP closed in 2002, it had achieved its objective of eliminating onchocerciasis as a public health and socio-economic problem,^5,11^ except in four river basins in Benin, Ghana, Guinea-Conakry and all of Sierra Leone. These special intervention zones (SIZs) received financial and technical support from 2003 to 2007 for ivermectin distribution and aerial or ground larviciding. When the SIZs were closed in 2007, the number of people treated was almost 6 million, with >75% treatment coverage in each SIZ.^12^

By the early 1990s, ivermectin MDA had been initiated in some African countries outside the OCP (e.g., Cameroon, Nigeria, Uganda) by Ministries of Health and NGDOs.^9^ The APOC was created in 1995 to support all African countries not part of the OCP in implementing a sustainable system for ivermectin MDA.^8,13,14^ In 2011, the population in the targeted meso- and hyperendemic areas (nodule prevalence among adult males >20%) was estimated at 86 million.^8,15^ To maximize treatment coverage and sustainability, the APOC adopted CDTI facilitated by the national health system and NGDOs.^8^ CDTI projects, each covering a limited geographic area within a country, allowed phased CDTI introduction. In 2015, more than 112.6 million people received ivermectin out of at least 185.9 million estimated to require treatment for onchocerciasis elimination (including hypoendemic areas).^16^

## From control as a public health problem to elimination of onchocerciasis in Africa

The APOC’s original goal was elimination of onchocerciasis as a public health problem,^17^ as it was uncertain whether onchocerciasis could be eliminated across Africa. In 2002, a conference concluded that ‘onchocerciasis is not eradicable using current tools due to the major barriers to eradication in Africa’.^18^

In 2005, three hyperendemic foci in Senegal and Mali where vector control had never been used were selected to evaluate whether CDTI can permanently interrupt *Onchocerca volvulus* transmission. The parasitological and entomological results suggested that 15–16 annual CDTI rounds in two foci and 17 years of biannual CDTI in one focus had eliminated infection and interrupted parasite transmission.^19^ The APOC’s objectives were consequently expanded to include ‘determination of when and where CDTI could be stopped and provision of guidance to countries to prepare for, effect and evaluate cessation of treatment’.^20^ The APOC evaluated infection prevalence in areas with long-term annual CDTI, which suggested that onchocerciasis may have been eliminated in many areas.^21^ Furthermore, the APOC consulted experts on critical issues such as delineation of transmission zones, determination of hypoendemic areas requiring interventions,^22^ identification of areas requiring intensified control to accelerate progress towards elimination and alternative treatment strategies (ATSs) (strategies other than annual CDTI) and prerequisites for their effective implementation.^23^

## ATSs

ATSs include complementary vector control, enhanced CDTI, community-directed treatment (CDT) with drug combinations or new drugs, and test-and-treat (TNT) strategies. All ATSs require significantly more human and financial resources and commitments at all levels of the health system than annual CDTI. Consequently, where CDTI will not achieve elimination by 2025 for programmatic reasons (reasons under the control of the health system and partners, as opposed to non-programmatic reasons such as hyperendemicity or loiasis co-endemicity), successful ATS implementation cannot be expected. In these areas, the first step has to be to optimize annual CDTI to achieve 100% geographic and ≥80% therapeutic coverage. Programme performance has to be evaluated against CDTI process indicators, including community participation, programme ownership, health education, mobilization, training of community drug distributors (CDDs), ivermectin supply, integration, coverage, monitoring and supervision. An important issue to address is systematic non-compliers, i.e., people who never or rarely participate in CDTI,^23^ who may represent a non-negligible proportion of the population (e.g., 15.5% within 10–12 years;^24^ 17.5% within 10 years^25^). The fact that compliance seems to be associated with individual perception of the CDTI programme (CDD commitment, MDA organization and perception of ivermectin effectiveness) needs to be taken into account.^26^

## Complementary vector control

Some OCP areas and the SIZs combined vector control and ivermectin distribution. The data from Guinea suggested that ivermectin distribution would allow reducing larviciding without a negative impact on progress towards onchocerciasis elimination.^27^ Combined approaches were also used in isolated foci in Uganda (Itwara and Kishoya-Kitomi foci)^28,29^ and Bioko Island.^30^

Given the costs, larviciding for vector elimination is not a realistic ATS. However, adding larviciding during peak transmission season to ivermectin treatment could significantly reduce biting, and thus reinfection rates. Such targeted larviciding was initiated in the Sanaga River in Cameroon.^31^ A number of factors need to be considered before launching vector control activities.^32^ Trapping of adult blackflies could reduce the biting rates, and thus transmission,^33^ but the impact of this strategy has never been assessed on *Simulium damnosum*.

## Enhanced CDTI

### Biannual or pluriannual CDTI

Studies of the effect of ivermectin treatment every 6 or 3 months suggest that more frequent treatment has a greater effect on macrofilarial reproductive capacity and longevity than annual treatment.^34^ Furthermore, more frequent treatment reduces skin microfilariae, and thus transmission. This strategy has contributed to onchocerciasis elimination in the small American foci. In Africa, some countries have introduced biannual CDTI in selected areas for different programmatic reasons, including a late CDTI start. Biannual treatment was introduced in 2006 in the isolated Abu Hamed focus of Sudan.^35^ In 2007, Uganda decided on biannual treatment in isolated foci where *Simulium neavei* elimination was not feasible.^36^ Biannual CDTI has also been used since 2012 (with vector control) in the large northern focus to combat nodding syndrome, a form of epilepsy associated with onchocerciasis.^37^ In 2009, Ghana adopted biannual CDTI in 40 of 73 districts following review of the epidemiological and entomological situation.^38^ In 2013, Ethiopia initiated biannual CDTI, especially in late-start CDTI projects.

There are, however, no data from comparative field studies that show the extent to which, all things being equal (pre-control endemicity, therapeutic coverage, etc.), biannual CDTI can accelerate progress towards elimination or enable elimination where annual CDTI might not. Modelling studies show that the effectiveness of biannual CDTI is as dependent on therapeutic coverage as that of annual CDTI and that the number of treatments to elimination is higher with biannual than annual CDTI.^39,40^ All activities to optimize annual CDTI need to be conducted as thoroughly for each biannual CDTI round to achieve high coverage in each round. Studies in different settings have shown that, except in loiasis co-endemic areas, compliance is mostly related to the perception of no treatment benefit and thus many systematic non-compliers will also not participate in biannual CDTI. Before deciding to switch from annual to biannual CDTI, countries need to ensure that the greater amount of human and financial resources required are committed.^38^

### Optimal timing of CDTI

In CDTI, populations decide on the period of ivermectin distribution, usually the dry (non-farming) season. In areas with highly seasonal transmission, this period may not be when CDTI would have a maximum impact on transmission. In some foci, changing the period of CDTI so that community microfilarial loads are lowest when vector abundance is highest can decrease the number of years to interruption of transmission. Models have shown that this effect is higher in areas with high pre-control endemicity (i.e., vector abundance) levels.^40^ Thus, in some foci, optimizing CDTI timing could accelerate elimination.

## CDT with drug combinations or new drugs

### Ivermectin–albendazole combination

A single ivermectin–albendazole combination treatment did not have a greater effect on *O. volvulus* than a single ivermectin dose.^41^ Results of a trial comparing both regimens administered annually or biannually over 2 years (ISRCTN50035143) are expected soon (http://www.dolf.wustl.edu/?page_id=777).

### Moxidectin

In two studies, a single 8 mg dose of moxidectin was superior to ivermectin in reducing and maintaining low *O. volvulus* microfilaridermia. Twelve months after moxidectin treatment, microfilaridermia was still lower or comparable to the nadir achieved 1 month after ivermectin treatment.^42–44^ A similar decrease in ocular microfilariae levels after moxidectin and ivermectin treatment contributes to a moxidectin safety profile compatible with CDT.^44–46^ EpiOncho modelling suggested that the number of years to reach thresholds for onchocerciasis elimination with annual moxidectin treatment is similar to that with biannual CDTI.^40^ Focusing programme resources on achieving high treatment coverage for annual moxidectin treatment may be more resource-effective than biannual CDTI with lower treatment coverage.^39^ A large study comparing multiple annual and biannual treatment with moxidectin and ivermectin is needed to better estimate the relative effect of moxidectin-based vs ivermectin-based strategies on microfilaridermia. Given moxidectin’s 20–30 day half-life,^42^ the cumulative effect of repeat dosing on macrofilarial reproductive capacity, viability and lifespan could be significantly higher than that recently identified for ivermectin.^34^ Until data on moxidectin’s effect on *L. loa* microfilariae are available, it has to be assumed that moxidectin could be used in *Loa* co-endemic areas only within a TNT strategy excluding people with high *Loa* microfilaremia. The not-for-profit organization Medicines Development for Global Health (MDGH; http://www.medicinesdevelopment.com/) has submitted a new drug application to the US Food and Drug Administration (FDA). FDA approval will trigger submissions to the regulatory authorities in African onchocerciasis-endemic countries. MDGH is preparing a study to determine a safe dose for 4- to 11-year-old children and is interested in sponsoring a large multiple treatment study, as suggested above. MDGH is also planning for affordable access to moxidectin for countries incorporating moxidectin into their control and elimination programmes.

## TNT strategies

A TNT strategy is ‘any strategy that requires diagnosis for infection and/or contraindications to treatment before a decision on who to treat with what regimen’ is made.^23^ The objective of TNT strategies is thus to diagnose individuals who need treatment and/or should be excluded from a particular treatment because of a risk of adverse reactions or no need for treatment. This can increase confidence in treatment relevance/benefit, particularly among asymptomatic individuals, and in treatment safety, particularly in areas with prior experience with severe adverse events (SAEs)—important prerequisites for increasing treatment coverage.^47–49^ Extensive mobilization needs to ensure participation of the whole community during each TNT campaign. This is all the more critical because the need for trained staff for testing requires the campaign period in a given village to be shorter than for CDTI. Consequently, successful TNT implementation requires significantly more resources and commitment of all partners and communities than annual or even biannual/pluriannual CDTI and its feasibility needs to be carefully examined. When this is ensured, TNT strategies can be designed for the specific epidemiological situation and public health objective(s) to be addressed and use drugs with a desirable efficacy profile but a treatment regimen or safety profile prohibiting CDT.

### Situations where TNT strategies could be applied

#### Loiasis co-endemic areas

**Figure 1.**
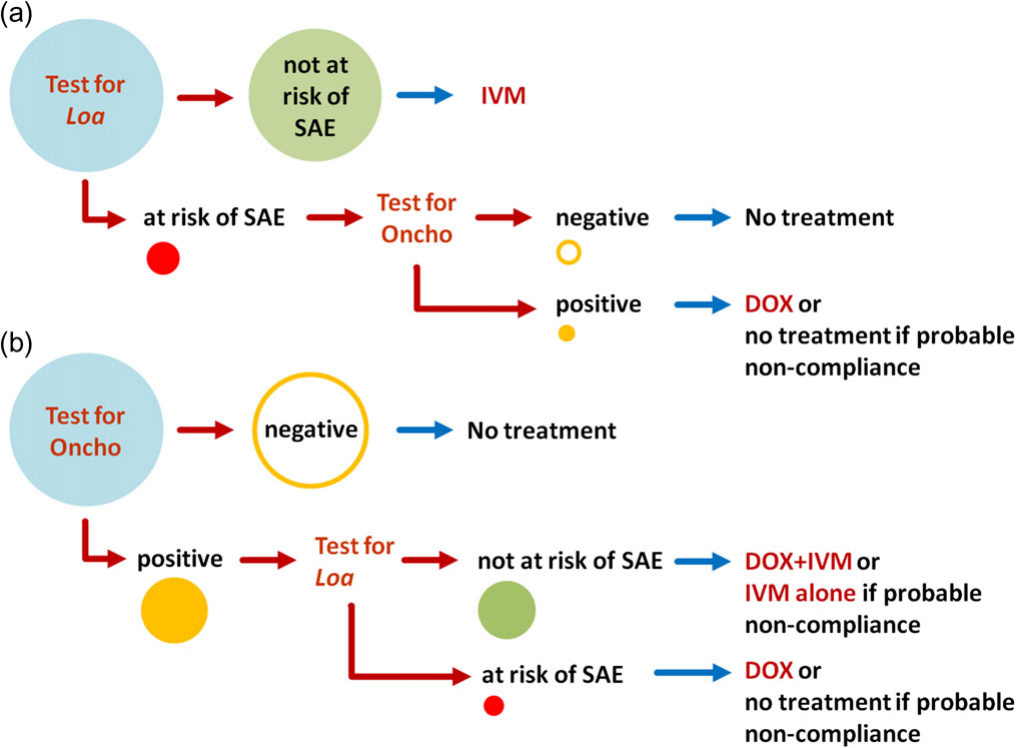
Decision trees for tests and treatment within the two possible TNT strategies: (a) Loa-first TNT; (b) Oncho-first TNT. The sizes of the circles correspond to the approximate relative size of the population to be tested or treated at each step in a community where loiasis coexists with hypoendemic onchocerciasis.

**Figure 2.**
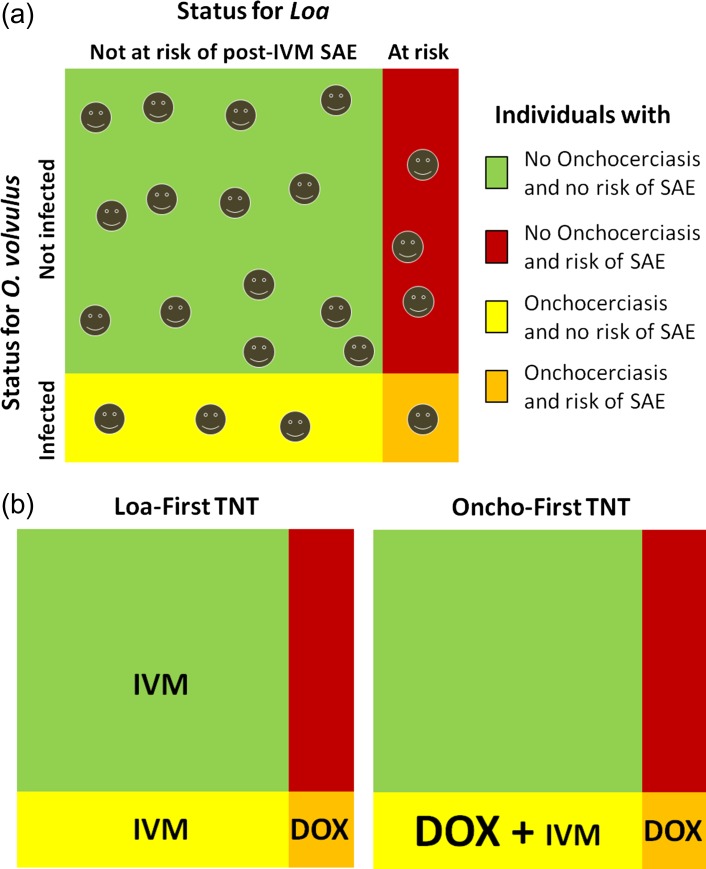
(a) Distribution of the individuals in a community where loiasis coexists with hypoendemic onchocerciasis according to their status regarding onchocerciasis (active infection or not) and their risk of developing a post-ivermectin SAE (individuals with >30,000 *Loa* mf/mL). (b) Treatment given (or not) to subjects in each of the four categories by TNT strategy used (Loa-first vs Oncho-first).

Individuals presenting with >30 000 *L. loa* microfilariae (mf)/mL of blood are at risk of post-ivermectin SAEs, including potentially fatal encephalopathy,^50^ due to ivermectin’s microfilaricidal effect on *Loa*. Where the *Loa* microfilaremia prevalence is >20%, 2–9% of adults have >30 000 mf/mL.^51^ In co-endemic areas where onchocerciasis is hypoendemic, the risk:benefit ratio prohibits CDTI implementation. Where onchocerciasis is meso-hyperendemic, CDTI can be implemented with special precautions to identify and manage SAEs,^52^ but fear of SAEs results in low coverage and high proportions of systematic non-compliers. Consequently, loiasis co-endemic areas are those where TNT strategies may be critical for onchocerciasis elimination. This includes hypoendemic areas (probably the priority), but also areas where CDTI has been ongoing for many years with a high proportion of systematic non-compliers. Two alternative TNT strategies could be applied. In the Loa-first TNT (Figure 1a), *Loa*-infected individuals at risk of SAEs are identified for exclusion from ivermectin (or other microfilaricidal) treatment, which can be given safely to the rest (usually >97%) of the population. Those at risk of ivermectin-related SAEs can then be tested for *O. volvulus* infection and, if indicated, treated with a macrofilaricidal drug (currently doxycycline is the only option). In an Oncho-first TNT (Figure 1b), *O. volvulus*–infected individuals are identified first and subsequently tested for *Loa* infection. Criteria for choosing between these strategies include the expected prevalences of *O. volvulus* and *Loa* infection and the respective cost-effectiveness (taking into account the material and personnel cost to apply the test, the number of persons to treat, the duration and the cost of treatment and the long-term impact on the *O. volvulus* reservoir). Carefully designed field studies are needed to compare the applicability and cost-effectiveness of both strategies. Figure 2 shows by TNT strategy the fractions of the population of an onchocerciasis hypoendemic community that would be treated with ivermectin and/or doxycycline.

#### Areas without loiasis

Where the prevalence of *O. volvulus* infection is low, the cost-effectiveness of an Oncho-first TNT strategy identifying and treating those infected should be compared with that of implementing/continuing CDTI. Such areas include hypoendemic areas as well as meso- or hyperendemic areas where many CDTI campaigns have reduced infection prevalence and a significant proportion of individuals that remain infected could be systematic non-compliers.

### Tests for TNT strategies

Calibrated thick blood smears, the current standard for *Loa* diagnosis,^51^ are not suitable for large-scale use. DNA- or antigen-detecting methods with good performance require laboratory equipment and cannot be applied at the point of care.^53^ The LoaScope, a smartphone-based video-microscope, allows the quantitation of *Loa* microfilaremia in the villages within 2 min after a fingerprick.^54^ The device was used in 2015 in a Loa-first TNT proof-of-concept study in the Okola health district (Cameroon), where in 1999 the first CDTI was interrupted after 23 SAE cases. Among 16 259 individuals tested, 340 subjects at risk of SAEs were identified and no SAEs were recorded in the rest of the population treated with ivermectin. The second TNT campaign in 2017 in the same district was equally successful and suggested that people treated once with ivermectin do not need to be retested during the subsequent year, which will dramatically reduce costs.

The standard for diagnosing patent infection with *O. volvulus* is identification of microfilariae in skin snips, which can be conducted in the villages, but this requires trained technicians and is relatively invasive. The OCP established the ‘DEC patch’ for surveillance of residual/new infections, detected via the localized skin reaction when microfilariae are killed under a patch containing diethylcarbamazine (DEC).^55^ A patch using transdermal delivery technology is now available for large-scale evaluation.^19,56^ Antibodies against the *O. volvulus* antigen OV16 can be detected by enzyme-linked immunosorbent assay. A field-suitable rapid diagnostic test (RDT) is undergoing large-scale sensitivity and specificity evaluation.^57^ It is unknown for how long OV16 antibodies are present after the last *O. volvulus* macrofilaria has died. The choice of the diagnostic test or sequence of different tests needs to consider the epidemiological control context, time and resources different tests require, their sensitivity/specificity, the risk–benefit profile of the drug(s) in false positives as well as the impact of not treating false negatives on achieving the elimination objectives.

### Treatments for TNT strategies

Currently, two drugs are available for TNT campaigns: ivermectin and the antibiotic doxycycline, which kills the *O. volvulus* symbiotic bacterium *Wolbachia*, resulting in sterilization (200 mg/d for 4 weeks or 100 mg/d for 5 weeks) and death of *O. volvulus* (200 mg/d for 6 weeks).^58–60^ Doxycycline has no microfilaricidal effect (and *Loa* has no *Wolbachia*) and consequently does not cause *Loa*-related SAEs. A study of community-directed doxycycline distribution supervised by the health system and involving approximately 13 000 subjects ≥12 years of age was conducted in Cameroon. The reported compliance with the 6-week course was very high (97.5% of participants complied by the end of 6 weeks).^61^ Outside a study context, effective systems to motivate, ensure and monitor compliance for 4–6 weeks, as well as effective pharmacovigilance, need to be in place. Compliance is all the more important given the lower efficacy of 3-week doxycycline (and 3-week minocycline) vs 4-week doxycycline.^62^ Pregnant women were excluded from the study in Cameroon based on self-reporting. The scientific rationale for regulatory label warnings against doxycycline use by pregnant women and children <8 years of age is being questioned.^63,64^ Until further evidence becomes available, pregnancy tests should be used to exclude pregnant women from doxycycline treatment, which is feasible within a TNT strategy. Doxycycline use needs to be consistent with global and national action plans on antimicrobial resistance.^65^

As part of a Loa-first TNT strategy, only the very few people excluded from ivermectin treatment and diagnosed as *O. volvulus* co-infected would require doxycycline treatment. Within an Oncho-first TNT strategy, those infected with *O. volvulus* can be treated with either ivermectin or doxycycline. If their number is low, doxycycline treatment of all may be feasible and more cost effective than repeated individual or mass ivermectin treatment. In all cases, individuals have to understand that compliance is crucial. When an individual’s determination/ability to comply with long-course treatment is doubtful, ivermectin should be given. A system to ensure and monitor complete compliance should be established with CDDs and the use of innovative tools (e.g., reminders by text messages).

Since doxycycline has no microfilaricidal effect, ivermectin should be given simultaneously to all those not at risk of SAEs to rapidly reduce *O. volvulus* microfilaridermia, the associated risk of ocular and skin manifestations and parasite transmission. People excluded from ivermectin treatment because of high *Loa* microfilaremia could be offered albendazole treatment to progressively reduce *Loa* microfilaremia.^66,67^

## Drugs and drug combinations in clinical or pre-clinical evaluation

Drug development is time consuming and risky, even when initial efficacy and/or safety data are available for the intended or other indications.^68^ Development of flubendazole, a promising drug candidate with proven macrofilaricidal activity,^69^ was discontinued due to non-clinical toxicology data (https://www.jnj.com/media-center/press-releases/janssen-discontinues-development-of-flubendazole-formulation-to-treat-onchocerciasis).

### Ivermectin–diethylcarbamazine–albendazole combination

A single ivermectin–diethylcarbamazine–albendazole (IDA) treatment may permanently sterilize or kill *Wuchereria bancrofti*.^70^ Therefore, IDA for onchocerciasis is being considered. The risk of general and irreversible ocular diethylcarbamazine-related SAEs in *O. volvulus*–infected individuals with high microfilaridermia requires ensuring a low microfilariae burden in each individual before IDA treatment.^71^ A TNT strategy with skin snips to quantify microfilaridermia and careful examination of ocular anterior and posterior segments could exclude individuals at risk of diethylcarbamazine-related SAEs from IDA treatment.

### Anti-*Wolbachia* compounds

Clinical studies are being conducted with antibiotics registered for non-filarial indications in humans and anti-*Wolbachia* activity in animal models to identify <14 or <7 day regimens for onchocerciasis and lymphatic filariasis (http://microbiology-bonn.de/immip/node/16; ISRCTN43697583, PACTR201608001754356). Following minocycline evaluation,^59^ trials are being considered with high-dose rifampicin.^72^

Tylosin is a veterinary antibiotic not approved for human use. The Anti-Wolbachia Consortium–AbbVie partnership evaluates tylosin analogues (TylAMac) for their anti-*Wolbachia* macrofilaricidal activity. In pre-clinical models, the two most potent analogues to date have antiparasitic efficacy, pharmacology and safety profiles suggesting oral treatment for ≤7 days might be efficacious and safe in humans (http://awol.lstmed.ac.uk/why-anti-wolbachia/tylosin-analogue-macrofilaricides-tylamac%E2%84%A2). A phase I study of the analogue ABBV-4083 will be initiated by the Drugs for Neglected Diseases initiative (DNDi) pending satisfactory results from ongoing toxicology studies (https://www.dndi.org/2017/media-centre/news-views-stories/news/filaria_rnd_status_feb_2017/). Corallopyronin A,^73^ methacycline, rifapentine and sparfloxacin^74^ depleted *Wolbachia* in animal filarial models and might be candidates for further development.

### Emodepside

Emodepside, approved in combination with praziquantel for veterinary use, has efficacy in animal filarial models. The DNDi has initiated a phase 1 study of its safety, tolerability and pharmacokinetics in healthy volunteers (NCT02661178). Studies in *O. volvulus* and *Loa-*infected individuals are planned for 2018. Emerging efficacy and safety profiles will determine whether emodepside development will continue and whether emodepside will be developed for CDT/MDA or TNT strategies.

### Other drugs

Other drugs are being evaluated for their antifilarial effects. Imatinib, a tyrosine kinase inhibitor approved for a number of indications in humans, has activity against *Brugia malayi* males, females, microfilariae and infective larvae (L3).^75^ The National Institute for Allergy and Infectious Diseases is planning a trial in *Loa-*infected individuals (NCT02644525). Auranofin, approved for use in rheumatoid arthritis, killed both *Brugia* spp. and *Onchocerca ochengi* adult worms *in vitro* and inhibited molting of L3s of *O. volvulus* with half-maximal inhibitory concentration values in the low micromolar to nanomolar range.^76,77^ Closantel, a veterinary drug, inhibited *O. volvulus* molting^78^ but had no macrofilaricidal effect and induced severe ocular adverse effects.^79^

## Tests under evaluation

While LoaScope performance is excellent, an RDT with similar performance but which is easier to apply is desirable for Loa-first TNT. Promising biomarkers of *Loa* possibly correlated with microfilarial density have been identified,^80^ and RDTs are being developed (http://www.intellectualventureslab.com/investigate/improving-sensitivity-to-flow-based-diagnostics). The not-for-profit biotech company Drugs & Diagnostics for Tropical Diseases has developed a *Loa* antibody detection test that may help further map loiasis distribution.^81^ Consideration needs to be given to the role these tests can play in TNT strategies.

The sensitivity of OV16-based tests could be improved by adding other antigens to the assay.^82^ Research for metabolites distinguishing serum of infected and uninfected individuals is ongoing.^83^ The urine concentration of N-acetyltyramine-O,β-glucuronide, a neurotransmitter-derived secretion metabolite in *O. volvulus*–infected individuals, is being evaluated as a potential diagnostic for active infection.^84,85^ Research on the diagnostic utility of parasite microRNA is ongoing.^86,87^ New RDTs usable for TNT strategies may be available in the coming years.

## Conclusion

Annual CDTI has achieved elimination of onchocerciasis as a public health problem in Africa. Alternative treatment strategies, ideally including new treatments and tests, can help eliminate onchocerciasis from most of Africa by 2025. A key requisite for onchocerciasis elimination is analysis of the situation in each focus to define the most cost-effective strategy to permanently interrupt parasite transmission. The National Onchocerciasis Elimination Committees being put in place will have a key role in determining the best approach in each focus. Since the APOC closure in 2015, the WHO-AFRO Expanded Special Project for Elimination of Neglected Tropical Diseases (ESPEN) coordinates technical advice and exchanges of experience between countries.
